# Trickle-Down Effects of Entrepreneurial Bricolage and Business Model Innovation on Employee Creativity: Evidence From Entrepreneurial Internet Firms in China

**DOI:** 10.3389/fpsyg.2021.801202

**Published:** 2022-02-02

**Authors:** Fei Hou, Ming-De Qi, Yu Su, Xiu-Xia Tan, Bin-Xin Yang

**Affiliations:** ^1^Institute of Advanced Studies in Humanities and Social Sciences, Beijing Normal University, Zhuhai, China; ^2^School of Management, Guangdong University of Technology, Guangzhou, China

**Keywords:** entrepreneurial bricolage, business model innovation, employee creativity, trickle-down, internet entrepreneurship

## Abstract

Although most existing studies have considered entrepreneurial bricolage as a means to overcome resource constraints in new ventures, few have explored the direct effects of entrepreneurial bricolage on employee creativity, particularly in the context of entrepreneurial internet firms. Drawing from multiple theories (i.e., social learning theory and social cognitive theory), this study proposes a cross-level mediation model for the trickle-down effects of entrepreneurial bricolage and business model innovation on employee creativity. By using a 2-wave longitudinal design, survey data were collected from multiple sources, including 49 leaders and 336 employees from entrepreneurial internet firms in China. Multilevel structural equation modeling (MSEM) was applied to analyze the cross-level mediation model. The results show that both entrepreneurial bricolage and business model innovation failed to significantly and positively direct employee creativity. Furthermore, entrepreneurial bricolage exerted a cross-level influence on employee creativity that was sequentially transmitted through between-level business model innovation and within-level creative self-efficacy. The theoretical and managerial implications of these findings are also discussed.

## Introduction

Bricolage, originally introduced by Lévi-Strauss (1967), has also been introduced and developed in the domain of entrepreneurship (Baker et al., 2003; Garud and Karnøe, 2003). Recently, in the domain of entrepreneurial research, bricolage has emerged as a central concept to better understand entrepreneur’s complex behavior and strategies in terms of development and utilization of resource (Kickula et al., 2018). Entrepreneurial bricolage (EB) is defined as “making do by applying combinations of the resources at hand to new problems and opportunities” (Baker and Nelson, 2005). It has been extensively researched in the domains of entrepreneurs and new ventures, and challenged the linear and causal approach in exploring entrepreneurial fashion of developing resources (Baker and Nelson, 2005; Kickula et al., 2018). To grow continuously and buffer environmental turbulence, EB is a strategic orientation that could actively and creatively aid new ventures to surmount resource constraints by reconfiguring existing resources (Garud and Karnøe, 2003; Baker and Nelson, 2005; Desa and Basu, 2013; Senyard et al., 2014; Yu et al., 2020). Amid the lack of resources common to new ventures, such ventures could be empowered by EB to survive and flourish by means of reusing and recombining resources at hand to overcome resource limitations (Baker and Nelson, 2005). Indeed, increasing empirical evidence has indicated that new ventures could have the capacity to manage resource limitations and improve performance though engaging in EB (Baker and Nelson, 2005; Di Domenico et al., 2010; Cunha et al., 2014; An et al., 2018). Hence, extant studies in the field of entrepreneurship mostly consider EB an approach to assist new ventures in filling resource gaps (Baker and Nelson, 2005; Desa, 2012).

Indeed, in addition to its effect on resource constraints, EB has other derivative effects. Some researchers have explored other effects of EB, particularly the effects of EB on knowledge generation. For example, some researchers have suggested that the concrete improvisational actions of bricolage can act as a way of experiential learning (Ferneley and Bell, 2006), that actions of bricolage can generate know-how (Andersen, 2008), and that new knowledge can be created by engaging in bricolage through blending parallel knowledge stocks (Boxenbaum and Rouleau, 2011). In this sense, the new knowledge created by bricolage could not only overcome resource inertia (Gilbert, 2005; Burgers et al., 2014) but also facilitate creativity and innovation for organizations (Andersen, 2008).

However, EB is a new and promising research field, and empirical research on EB is in its infancy.

In particular, the relationship between EB and employee creativity has rarely been explored. In current rapidly changing and challenging business environments, work in organizations has become increasingly knowledge-based and dynamic, and thus creativity has been increasingly regarded as a key catalyzer to trigger employees’ performance and success and to build and sustain the core competence of organizations (Anderson et al., 2014). In this respect, identifying factors that could lead to employee creativity has generated a growing stream of research (e.g., Zhou and George, 2001; Hülsheger et al., 2009; Xu et al., 2019). Prior studies have developed an intensive interest in exploring how leadership influences employee creativity (Hennessey and Amabile, 2010). Similarly, scholars have come to share a strong interest in understanding how EB fosters employee creativity. In this respect, it is of both theoretical and practical importance to investigate the influence of EB—a kind of resource-recombining behavior by new ventures (Baker and Nelson, 2005)—on employee creativity as well as the underlying mechanism for this influence. Hence, the first research motive of this study is to explore whether and how EB fosters employee creativity in new ventures.

Entrepreneurial bricolage has been introduced into a range of fields, and it has been considered beneficial for explaining various organizational phenomena. For instance, it has been applied to illustrate the activities of entrepreneurs (Garud and Karnøe, 2003) and to explicate why entrepreneurs have the capability to create something from nothing (Baker and Nelson, 2005). In this sense, according to White and Lean (2008), the atmosphere or culture set by an organization’s leaders or top managers, regardless of the management level, could have an effect on followers’ behaviors. In the leadership literature, considerable empirical evidence has supported the cascading effect of role modeling from high-level leaders on their followers’ responses (e.g., Mayer et al., 2009). Thus, Liu et al.’s (2012) study described an example that denoted how a department leader’s abusive supervision subsequently triggers and influences employee creativity. This trickle-down effect could be reasonably explained by drawing from social learning theory (SLT), whereby the behavior of high-level leaders may be imitated and displayed by low-level followers. As such, to elaborate on the idea that EB might have a trickle-down effect, the second research motive of this study is to test a trickle-down model to explore whether EB at the organizational level engenders creative behavior at the individual level.

A primary concern for organizations is to nourish the employee creativity that produces innovative results. According to Shalley et al. (2004), the growth of creativity among employees could be attributed to factors at the individual level and the context in which they work. In this respect, leader behavior has been regarded as a key factor in influencing a work context that could foster creativity among employees (Amabile et al., 2004). Empirical evidence has shown that leaders could have a contextual effect on employees’ performance, which yields creative outcomes (Shalley and Gilson, 2004). Hence, a large body of prior research has focused on nourishing employee creativity through a specific leadership style (e.g., Mumford et al., 2002; Reiter-Palmon and Illies, 2004; Wang et al., 2013).

A business model is key in influencing the competitive advantage of firms (Chesbrough, 2010), and thus, business model innovation (BMI) plays a critical role in building a sustainable competitive advantage (Johnson et al., 2008; Demil and Lecocq, 2010). Based on the reasoning above, as a contextual factor, BMI might foster a supportive innovative climate (e.g., Charbonnier-Voirin et al., 2010; Wang et al., 2013) and mobilize the necessary organizational resources to motivate employees to engage in creative behaviors. Although prior studies have shed light on BMI, they have been predominantly concerned with its antecedents (e.g., Guo et al., 2016), thereby ignoring its consequences. Furthermore, studies on the consequences of BMI have primarily focused on organizational-level outcomes (e.g., firm performance and success; Giesen et al., 2007).

Prior multilevel studies have provided empirical evidence that organizational-level variables, including leadership style (Jaiswal and Dhar, 2015), coworker support (Hon, 2011), and support for innovation (Chen et al., 2013), positively relate to individual-level creativity. Indeed, these findings indicate that it is important to consider BMI from a multilevel perspective. However, it is still unclear how an innovation climate initiated by BMI influences employee behavior, which limits a clear understanding of BMI as a cross-level phenomenon. Hence, there is a strong theoretical linkage between organizational-level BMI and individual-level creativity. Theoretical advancements and refinements in the creativity literature might be hindered by a lack of empirical tests of these multilevel propositions. Correspondingly, the third motive of this study is to further explore the individual-level consequences of BMI.

In addition, previous research has revealed that individuals with a high level of creative self-efficacy (CS) typically generate creative ideas (Tierney and Farmer, 2002, 2011; Gong et al., 2009). CS research has provided empirical evidence that CS plays a mediating role in fostering creativity among employees (Gong et al., 2009). Moreover, although the reciprocal relationship between CS and individual creativity has been highlighted by Bandura (1997), there is little research that explores how organizational-level variables motivate individuals to seek guidance in displays of creative performance through the cross-level mediating effect of CS. According to Chen and Kanfer’s (2006) foundational theorizing, there are motivational processes at both the individual and organizational levels. Hence, organizational-level EB and BMI might enhance the level of CS among employees to generate their creative behaviors. As such, drawing from Bandura’s (1986) social cognitive theory (SCT), the present research attempts to bridge this knowledge gap by exploring the cross-level mediating effect of CS in the relationship between organizational-level variables (e.g., EB and BMI) and individual-level outcomes (e.g., EC).

Consequently, by building and testing a multilevel theoretical model, this study aims to make unique contributions to the literature on EB and creativity in the following meaningful ways: First, this study contributes to the creativity literature by exploring the top-down effect of organizational variables (EB and BMI) on individual creative behaviors. Based on a trickle-down model, this study offers empirical evidence for whether organizational-level variables (e.g., EB and BMI) could exert a top-down effect on individual creative behaviors (e.g., EC), which could advance our knowledge of the cross-level relationship between EB/BMI and EC. To the best of our knowledge, this study is the first empirical investigation of the cross-level influences of EB and BMI on individual creative behavior. In doing so, it substantially contributes to the theoretical understanding of the implications of EB and BMI across different levels of analysis.

Second, this study takes a rather nuanced approach to better understand how organizational-level EB manifests in individual-level EC. Given that a team-level proactive personality could influence individual behaviors through group dynamics (Fuller and Marler, 2009; Wang et al., 2017; Xu et al., 2019), it follows that EB will affect BMI, thereby fostering an organizational climate for innovation and subsequently facilitating individual creative behaviors. Although a positive correlation between EB and BMI has been identified (Guo et al., 2016), few studies have explored whether EB affects followers’ creative behaviors through its influence on BMI. Hence, this study is the first to elucidate the underlying mechanism through which EB influences EC, contributing to the creativity literature by illustrating a new and more fine-grained picture of the cross-level mediating effect of BMI in the EB-EC link.

Third, this study explores whether EB and BMI have cross-level influences on creative efficacy beliefs and subsequent engagements in individual creative behaviors. By introducing CS as an individual-level underlying mechanism that drives workplace creativity, the cross-level mediation mechanism in the relationship between EB/BMI and EC will be identified. Hence, this study enriches the growing research on the cross-level influences of organizational variables (e.g., EB and BMI) on employee creativity by revealing the mediating role of CS.

**FIGURE 1:**
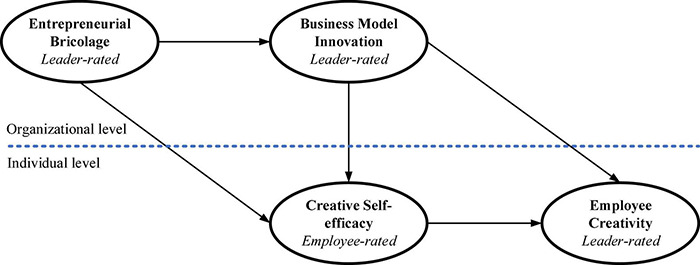
Hypothesized model.

Finally, this study complements previous research by indicating the effects of EB on internet entrepreneurship. Although prior studies on the effects of EB have often used entrepreneurs and new entrepreneurial firms as their research contexts (e.g., Baker and Nelson, 2005; Cunha et al., 2014; Senyard et al., 2014), few have explored the effects of EB on internet entrepreneurship. Among all types of new ventures, internet ventures are more apt to build core competencies by enhancing creativity and innovation to overcome resource constraints. Indeed, EB could directly benefit entrepreneurial internet firms that strain to overcome resource inertia by creating a new way for them to apply existing resources, regardless of the overall availability of resources (Levi-Strauss, 1966). However, to the best of our knowledge, few studies have examined the effects of EB on internet ventures. Our theoretical model is presented in Figure 1.

## Theoretical Orientations

### Top-Down Influence of Entrepreneurial Bricolage on Employee Creativity

According to Baker and Nelson (2005), bricolage is defined as “making do by applying combinations of the resources at hand to new problems and opportunities.” There are three key elements in this definition. The first element is “making do,” which indicates “a bias toward action and active engagement with problems or opportunities rather than lingering over questions of whether a workable outcome can be created from what is at hand” (Baker and Nelson, 2005). The second element is the “combination of resources for new purposes,” which implies “the combination and reuse of resources for different applications than those for which they were originally intended or used” (Baker and Nelson, 2005). The third element is using “resources at hand,” which involves “resources that are available very cheaply or for free” (Baker and Nelson, 2005).

Bricolage has been successfully introduced and applied in the research domain of entrepreneurship (Baker et al., 2003; Garud and Karnøe, 2003), and it could aid interpretations of various entrepreneurial phenomena. EB has been considered to play a significant role in early stages of ventures, and resource development for entrepreneurs evolving in resource constrained environments (Baker and Nelson, 2005; Kickula et al., 2018). In particular, EB has been utilized to describe entrepreneurs’ activities (Garud and Karnøe, 2003). In this respect, EB has been applied to explain why some entrepreneurs are able to create something from nothing (Baker and Nelson, 2005). Sarasvathy (2008) noted that EB, the concept of making do with resources at hand, could be employed to depict effectual entrepreneurship.

Creativity has been defined as the development of novel and useful ideas regarding products, practices, services, or procedures (Amabile et al., 1996). In the rapidly changing era of the knowledge economy, creativity has been increasingly regarded to play a dominant role in the survival and competitiveness of organizations. Given that employee creativity has traditionally been considered to be influenced by leaders (e.g., Amabile et al., 2004; Liao et al., 2010), limited research has explored the effect of leader behavior, in the form of EB, on creativity.

Indeed, how leaders at hierarchical levels affect employee behaviors is a critical and controversial topic. Although some studies have demonstrated that immediate supervisors might have a greater impact on employee behavior than others, other studies have indicated that top leaders or managers who reflect an organizational image should exert a greater effect on employee behavior (e.g., Schneider et al., 1995). For instance, according to Basford et al. (2012), employees’ intentions to stay with a firm could be significantly influenced by senior management’s support. In line with this logic, when acting as representatives of organizations, top leaders or managers are more likely to have greater influence on their followers and to play a more significant role in enhancing follower creativity.

Drawing from SLT (Bandura, 1971), observing and modeling the behavior, attitudes, and emotional reactions of others can play a significant role in how individuals think and behave. Most displayed individual behaviors could be learned, either deliberately or inadvertently, through the influence of a role model (Bandura, 1971). As such, role modeling has been regarded as an important mechanism for leadership learning. Indeed, most leadership topics (e.g., charismatic leadership, transformational leadership, and ethical leadership; Bass et al., 1987; Mayer et al., 2009) have concerned role modeling. For instance, according to Brown et al. (2005), by means of intentionally acting as role models, leaders might win over their followers. Hence, extending the similar reasoning of the trickle-down effect of leaders’ behavior (e.g., Mayer et al., 2009; Liu et al., 2012), this study suggests that leaders or entrepreneurs engaging in EB are more likely to influence their followers’ creative behaviors.

Bandura (1969) further contended that model characteristics are a key factor that could influence responsiveness to role modeling. Compared to models with low status and power, models with high status and power could exert more influence on followers to imitate them. SLT has shown that models with qualities of high competence, status, prestige, and power are more likely to be imitated by followers than others (Bandura, 1971). Following this logic, in the entrepreneurship context, since entrepreneurs take charge of the allocation of venture resources and engage in EB, they usually possess a high status and great power and are respected by their followers. As a result, such followers are more likely to be inclined to take them as role models and then to observe and imitate their behaviors. Accordingly, when individuals perceive heterogeneous combinations of resources initiated by their role models (e.g., entrepreneurs), creative outcomes could be generated (Penrose, 1959).

Thus, having drawn the above discussions about the effects of EB on employee creativity, this study proposes the following:

**Hypothesis 1:** Entrepreneurial bricolage is positively associated with employee creativity.

### Multilevel Mediation Through Business Model Innovation

Based on the definition of Amit and Zott (2001), the business model comprises “the content, structure, and governance of transactions designed so as to create value through the exploitation of business opportunities.” Business models could build competitive advantages for firms (Zott and Amit, 2007; Johnson et al., 2008; Ahlstrom, 2010). However, market changes can quickly make a successful business model less profitable or even obsolete (Johnson et al., 2008; Sosna et al., 2010). Moreover, business models might be imitated by other firms when firms observe successful business models and then introduce them into their own businesses (Casadesus-Masanell and Zhu, 2013). Amid increasingly fierce competition and fast-changing technology, the capacity to reinvent a business model plays an important part in both attenuating obsolete business models and sustaining firm performance (Demil and Lecocq, 2010). Thus, BMI plays a critical role in building a sustainable competitive advantage for firms (Johnson et al., 2008). Empirical evidence supporting the positive linkage between BMI and firm performance has been provided (Giesen et al., 2007).

Due to the importance of BMI, prior studies have developed vital research, mostly by exploring the antecedents of BMI (Chesbrough, 2010; Zott et al., 2011; Amit and Zott, 2012). Based on theoretical analyses and in-depth case studies on the determinants of BMI, some works have shown that BMI is a process of experimentation (e.g., Hayashi, 2009; McGrath, 2010; Sosna et al., 2010). In this respect, EB has been considered a key constituent action of the experimentation process of BMI. According to Baker and Nelson (2005), EB needs to experiment with new alternatives to reuse and recombine resources, deal with new problems and identify opportunities. Innovating the content, structure, and governance of transactions and taking advantage of new opportunities have been shown to contribute to BMI (Zott and Amit, 2010). Through reusing and recombining the resources at hand to cope with new problems and opportunities, EB can aid a venture firm in innovating new transactional content, structure, and governance as well as in identifying new opportunities (Baker and Nelson, 2005), all of which, accordingly, facilitate BMI.

Additionally, this study argues that BMI can foster individual creative behaviors. One possible reason for this is that BMI can significantly generate and foster an organizational climate for risk taking or innovation, and thus, a supportive innovation climate can promote employees’ creative behaviors (Jung et al., 2003; Černe et al., 2013). More specifically, a venture firm with a high BMI will usually create an “innovative or creative” climate that spreads across organizations or departments. This climate signals the organizational expectations for potential creativity-related behaviors to members and an organizational willingness to provide support for innovation, thereby inspiring members to take risks and sponsor innovations (Wang et al., 2013). Accordingly, such an innovation-oriented work climate could set clear expectations and norms and provide accessibility to organizational resources and support, which give organizational members more possibilities for creative outcomes. In the innovative climate, members are encouraged to value experimentation, tolerate occasional flaws, engage in risk-taking behaviors, and be more motivated to use a creative approach at work, facilitating and shaping the creative behaviors of employees. In addition, to access the support from their organization or group, members might adopt the behaviors expected by this organizational innovation-oriented climate (Dragoni, 2005).

Thus, taking the above arguments together, this study suggests that BMI is a conduit through which EB realizes its contributions to EC. In other words, BMI represents an organizational-level activity that a venture firm could take to expedite the effects of EB on EC. Hence, this study proposes the following:

**Hypothesis 2**: BMI mediates the cross-level relationship between entrepreneurial bricolage and employee creativity.

### Multilevel Mediation Through Creative Self-Efficacy

Based on the definition of CS by Tierney and Farmer (2002), CS represents the degree of an individual’s belief in his or her capacity to generate creative outcomes. Drawing from self-efficacy theory (Bandura, 1997), the term CS originates from the concept of an individual’s belief about self-capacity on the basis of one’s knowledge, skill, and ability, which are the prerequisites for specific creative performance. Recent creativity studies have intensified the role CS plays in mobilizing employees’ creative efforts by determining EC in organizational contexts (Gong et al., 2009; Diliello et al., 2011; Tierney and Farmer, 2011; Wang et al., 2013). Furthermore, prior studies have explored the mediating role of CS in the relationship between transformational leadership and EC (Gong et al., 2009; Wang et al., 2013; Mittal and Dhar, 2015). However, little interest has been shown in exploring the mediating role of CS between EB and EC, and thus, it is useful to investigate the mediating effect of CS on this relationship.

According to Duymedjian and Rüling (2010), bricolage has been implicitly considered a special behavioral process to reconstruct the resources at hand. Thus, bricoleurs could create something from nothing through experimenting with a variety of possible resources (Cunha et al., 2014). In the process of resource reconstruction, deeper level experimental knowledge regarding the resources at hand and their usages can be developed (Cunha, 2005). Indeed, according to the original research of Levi-Strauss (1966), bricolage has been depicted as a resource-learning approach to produce new knowledge. Furthermore, the knowledge generated through bricolage has an individual history and heterogeneous nature and is sometimes even integrated into the identity of the bricoleur (Duymedjian and Rüling, 2010).

Moreover, when regarded as an interaction experience and a kind of trial-and-error learning, bricolage can aid organizations in producing new experience-based knowledge (Duymedjian and Rüling, 2010). As a result, the new knowledge generated by bricolage could generate unique perceptions of the surrounding environments (Cunha et al., 2014) and services that the resources of a firm could render (Penrose, 1959; Cunha et al., 2014) to shed new light on how to recombine different knowledge elements to produce new products and services (Boxenbaum and Rouleau, 2011).

Compared to individual activity, bricolage at the organizational level represents a collective action of experiential learning that involves a process of “give and take” through dynamic interactions among organizational members (Baker and Nelson, 2005). For example, Garud and Karnøe (2003) noted that these interactions could take place between researchers and producers, between producers and users, between designers and workers, and between policy-makers and the markets that they regulate. As such, these multiple interactions could generate more opportunities for organizations to obtain new knowledge for resource reconstruction. However, not all organizations will fully exchange multiple learning opportunities. As a result, in the entrepreneurial context, entrepreneurial firms are more likely to encourage their employees to create and use new knowledge to “think outside the box” (Baker and Sinkula, 1999; Nasution et al., 2011), entailing that they might benefit more from the collective nature of the EB learning process.

Individuals query information from their workplaces to enhance creative-specific self-efficacy, and thus contextual factors could influence CS (Tierney and Farmer, 2002). Thus, given that the organizational-level learning initiated by EB is contextual, individuals could benefit from this working context by consolidating previously scattered experiential knowledge, which might enhance individuals’ beliefs regarding their creative activities. In the context of entrepreneurship, EB has substantial potential to foster an organizational climate of learning that is regarded as a specific working context, which could motivate members to share knowledge and experiences and to pool diverse resources to facilitate creative performance by offering support and encouragement. Accordingly, employees are more likely to feel confident in generating new ideas and displaying creativity. Hence, this study argues that this working context, in the form of an EB-initiated learning climate, could be beneficial to employees’ creative-specific efficacy.

In addition, the relationship between CS and EC has been supported by previous empirical evidence (e.g., Tierney and Farmer, 2011). More specifically, CS has been shown to encourage individuals to exert the effort needed to generate creative ideas by influencing their creative expectations (Tierney and Farmer, 2002; Gong et al., 2009; Wang et al., 2014). Furthermore, according to Amabile (1996), creativity generation usually requires individuals to take risks; thus, it is necessary for them to obtain sufficient confidence to overcome problems and challenges. In this regard, CS could provide internal and sustaining support to encourage individuals to exert the effort needed for creativity (Tierney and Farmer, 2004; Baer et al., 2008).

Thus, taking the above arguments together, this study suggests that EB, as a working context, can effectively foster employees’ CS, which, in turn, positively influences employee creative performance. Hence, this study proposes the following:

**Hypothesis 3**: Creative self-efficacy mediates the cross-level relationship between entrepreneurial bricolage and employee creativity.

Moreover, in line with the reasoning regarding the influences of BMI, an innovation-oriented work climate fostered by BMI might enhance employees’ CS by setting creative expectations and norms and offering easy accessibility to organizational resources and support. According to Williams and Foti (2011), a supportive organizational climate could aid employees in sustaining their creative paths and mobilizing their creative potentials to produce creative outcomes. The existence of such a climate at the organizational level could facilitate an edge that promotes employees’ confidence in producing new ideas. Hence, this working context, in the form of an innovation-oriented climate, could be beneficial to employees’ creative-specific efficacy.

Taking our theoretical development for Hypotheses 2 and 3 and the above arguments together, EB might enhance BMI (e.g., as a proximal mediator linking EB to itself) and therefore strengthen employees’ CS (e.g., as the distal mediator), which, in turn, leads to the generation of EC. Hence, this study proposes the following:

**Hypothesis 4.** Business model innovation and creative self-efficacy sequentially mediate the cross-level relationship between entrepreneurial bricolage and employee creativity.

## Research Design and Methods

### Sample and Procedures

According to the extant literature, the information technology (IT) industry and entrepreneurial internet firms are especially concerned with innovation, and employees’ performance and success can be triggered mainly by creativity (e.g., Cooper, 2000; Yeh, 2004; Xu et al., 2019). Accordingly, our survey team recruited leaders and employees from entrepreneurial internet firms in China.

Our survey area is the Pearl River Delta region of Guangdong Province, which is the fastest growing region in southern China, possesses a strong entrepreneurial and creative atmosphere and generates a large number of entrepreneurial practices and creative activities. Specifically, the sampled entrepreneurial internet firms come from the entrepreneurial incubators, entrepreneurial and creative parks, and maker spaces (e.g., Southern Software Park, Jinjia Creative Valley, and V12 Pioneer Park) that are mainly located in Guangzhou, Shenzhen, and Zhuhai.

For a higher response rate and accuracy of survey data, our research team first directly contacted directors of entrepreneurial and creative incubators and parks to ask for their assistance, and then questionnaires were distributed to the leaders and employees of the sampled entrepreneurial internet firms. In this survey, the term leaders refers to entrepreneurs or at least one top manager of each sampled firm, and the term employees refers to workers directly supervised by the survey’s leaders. Employees were asked to provide employee identification numbers to match their responses. After matching the data, 395 employees within 49 entrepreneurial internet firms completed the survey, and a dyad of 336 employee-leader matched datasets comprised our final sample, demonstrating a response rate of 85.1%.

In the survey, our survey team informed participants about the research purposes, managerial implications of the study, importance of a careful response to each survey item, and assured them of the confidentiality of their responses. Two types of questionnaires (including leader surveys and employee surveys) were administered at two instances (Time 1 and Time 2) with a 2-month interval and were distributed to the leaders and their employees.

For the employee survey, at Time 1, employees completed demographics and CS measures. The sample included 56.10% males and 43.90% females. Regarding education level, 86.06% had a bachelor’s degree, 11.50% had a master’s degree, and 2.44% had a doctoral degree. In the case of the leader survey, at Time 1, leaders were asked to provide information about their venture size and tenure and to complete EB and BMI measures. At Time 2, leaders were asked to evaluate their employees’ creativity (EC). Leader demographics consisted of 60.63% males and 39.37% females. The average venture size was 8.16 (SD = 1.056), and the average venture tenure was 1.99 (SD = 1.106).

However, the issue of potential common method basis was minimized by using different sources of survey data (e.g., leader surveys and employee surveys). Harman’s one-factor test was conducted to further check common method bias. Based on the principal component factor method, the results indicated that 37.60% of the variance was explained by the first factors in the model. Hence, common method bias was not an issue.

### Measures

The scales were originally developed in English and then translated to Chinese by using the back-translation procedure (Brislin, 1980). Unless otherwise indicated, all items were rated on a 7-point Likert-type scale (1 = strongly disagree; 7 = strongly agree).

#### Entrepreneurial Bricolage

Entrepreneurial bricolage was measured with three items of the scale adopted from Baker and Nelson (2005). The top managers of the sample ventures were asked to report how extensive they felt EB was at their ventures. A sample item was “Applying combinations of resources at hand to create new products or services.” According to Geldhof et al. (2014), conflation in reliability estimates at the within level and between level can be prevented by a multilevel confirmatory factor analysis (MCFA) since it can decompose measurement model parameters into level-specific parts. Hence, following the recommendation of Koopmann et al. (2016), this study applied the MCFA approach to assess the model constructs’ Cronbach’s alpha at the within and between levels. The organizational-level Cronbach’s alpha based on the MCFA analysis for this scale was 0.865.

#### Business Model Innovation

According to extant studies (e.g., Zott and Amit, 2008; Guo et al., 2016), BMI was measured with a 7-item scale. Top managers of sample ventures were asked to rate how extensive they felt the level of BMI was at their ventures. A sample item was “Our business model offers new combinations of products, services and information.” The organizational-level Cronbach’s alpha for this scale was 0.828.

#### Creative Self-Efficacy

Creative self-efficacy was measured with three items of the scale developed by Tierney and Farmer (2002). Employees of sample ventures were asked to report the extent to which the statements accurately described their efficacy with respect to creative work. A sample item was “I have confidence in my ability to solve problems creatively.” Based on a one-way ANOVA, the results showed that CS possessed a high between-level variation and within-level agreement [*F* = 2.861, *p* < 0.05; ICC (1) = 0.24; ICC (2) = 0.85]. Furthermore, an MCFA analysis (Geldhof et al., 2014) indicated that the Cronbach’s alpha was 0.849 at the between-team level and 0.846 at the within-team level, demonstrating that the measure was reliable at both between and within levels.

#### Employee Creativity

Employee creativity was measured with four items of the scale developed by Tierney and Farmer (2011). The top managers of the sample ventures were asked to rate how extensive they felt the individual creativity of each employee was. A sample item was “This team member identifies opportunities for new ways of dealing with work.” Based on a one-way ANOVA, the results showed that CS possessed a high between-level variation and within-level agreement [*F* = 6.584, *p* < 0.001; ICC (1) = 0.49; ICC (2) = 0.84]. Furthermore, an MCFA analysis (Geldhof et al., 2014) indicated that Cronbach’s alpha was 0.918 at the between-team level and 0.810 at the within-team level, demonstrating that the measure was reliable at both between and within levels.

#### Controls

According to extant studies, to prevent research bias, the following variables were taken at two levels as controlling variables. Employee gender and educational level were included as controlling variables at the individual level (e.g., Gong et al., 2009; Richter et al., 2012; Shin et al., 2012). Leader gender, venture tenure (in years) and venture size (total number of employees) were included as controlling variables at the organizational level (Guo et al., 2016).

### Analytical Strategy

Due to the nested survey data (i.e., employee responses were nested within their ventures), multilevel structural equation modeling (MSEM) was recommended to test all our hypotheses (Preacher et al., 2010). Compared with hierarchical linear modeling (HLM), the MSEM approach could more effectively prevent potential problems of conflated within- and between-level relationships and assess cross-level indirect effects by decomposing variances into components at the between and within levels (Zhang et al., 2009; Preacher et al., 2010).

For the cross-level indirect effects proposed in this study (i.e., 2-1-1, 2-2-1, and 2-2-1-1), the MSEM approach could simultaneously evaluate the organizational-level relationship between EB and BMI, the top-down relationship between BMI and CS, and the individual-level relationship between CS and EC. Thus, according to the recommendation of Zhang et al. (2009), the cross-level indirect effects were examined by multiplying the path coefficients among the latent predictor (EB), latent mediator (BMI), latent group mean of the mediator (CS), and the latent group mean of outcome (EC). Hence, the point estimates and standard errors of cross-level indirect effects were obtained on the basis of unstandardized coefficients of proposed multilevel model paths. Furthermore, a Monte Carlo simulation with 20,000 replications was conducted to test the 95% bias-corrected confidence interval (CI) around the cross-level indirect effects (Preacher et al., 2010).

In this study, all analyses were conducted by using Mplus 8.0 (Muthén and Muthén, 1998–2010) with a robust maximum likelihood (MLR) estimation. Following the recommendation of Hu and Bentler (1999), root mean square error of approximation (RMSEA), Tucker–Lewis Index (TLI), and comparative fit index (CFI), the standardized root mean square residual for the within-level (i.e., individual-level) model (SRMR-within; Hu and Bentler, 1999), as well as the standardized root mean square residual for the between-level (i.e., team-level) model (SRMR-between; Hsu et al., 2015), were adopted to evaluate model fit. Furthermore, scaled chi-square difference testing was employed to compare alternative rival multilevel models.

## Results

### Descriptive Statistics

Table 1 presents the descriptive statistics and correlations among model constructs. Notably, as shown in Table 1, education level, the individual-level controlling variable, was not significantly related to substantive variables. According to Becker’s (2005) recommendations, control variables should be excluded when they are not associated with dependent variables to prevent reduced statistical power and increased Type II errors. Hence, educational level was excluded from any subsequent analysis.

**TABLE 1:** Means, standard deviations, and inter-correlations of variables.

Variable	*M*	SD	1	2	3	4	5
***Individual level***							
1. Gender T1	1.440	0.497	1				
2. Education level T1	1.160	0.432	0.136*	1			
3. Creative self-efficacy T1	5.508	0.819	0.154*	0.115	**0.789**		
4. Employee creativity T2	5.464	0.815	0.161*	0.124	0.664**	**0.785**	
***Organizational level***							
1. Leader gender T1	1.39	0.489	1				
2. Venture tenure T1	1.99	1.106	0.057	1			
3. Venture size T1	8.16	1.056	0.093	0.440**	1		
4. Entrepreneurial bricolage T1	5.831	1.140	0.016	0.012	0.203**	**0.886**	
5. Business model innovation T1	5.753	0.857	0.195	0.077	0.233**	0.620**	**0.773**

*Individual level N = 336; organizational level N = 49. Gender was dummy-coded (0 = female, 1 = male). Education level was categorically measured (1 = bachelor degree, 2 = master degree, 3 = doctoral degree). Venture tenure was categorically measured (less than 1 year as 1, 1–3 years as 2, 3–5 years as 3, 5–8 years as 4). Venture size was categorically measured (less than 10 as 1, 10–50 as 2, 50–100 years as 3, over 100 as 4). Bold value indicates the square root of each latent variable’s average variance extracted (AVE). T1, Time 1; T2, Time 2.*

*p < 0.05.

**p < 0.01.

### Convergent and Discriminant Validity

A confirmatory factor analysis (CFA) was used to assess the reliability, convergent validity, and discriminant validity of the model constructs. As shown in Table 2, the model constructs demonstrated a high internal consistency in terms of their Cronbach’s alphas and composite reliability (CR). All factor loadings for model constructs were statistically significant (for EB: 0.822–0.931; for BMI: 0.725–0.834; for CS: 0.754–0.809; for EC: 0.740–0.812) and all were over the recommended criteria of 0.700, indicating acceptable convergent validity. In addition, all the measurements possessed adequate item reliability in terms of square multiple correlation (SMC) values.

**TABLE 2:** Overall reliability of the constructs and factor loadings of indicators.

Construct (source)	Items	Factor loading	SMC	Cronbach’s alpha	CR	AVE
**Entrepreneurial bricolage** (Baker and Nelson, 2005)	EB1	0.931	0.867	0.857	0.916	0.785
	EB2	0.901	0.812			
	EB3	0.822	0.676			
**Business model innovation** (Zott and Amit, 2008; Guo et al., 2016)	BMI1	0.834	0.696	0.890	0.913	0.599
	BMI2	0.783	0.613			
	BMI3	0.782	0.612			
	BMI4	0.778	0.605			
	BMI5	0.776	0.602			
	BMI6	0.736	0.542			
	BMI7	0.725	0.526			
**Creative self-efficacy** (Tierney and Farmer, 2002)	CSE1	0.809	0.654	0.796	0.831	0.622
	CSE2	0.802	0.643			
	CSE3	0.754	0.569			
**Employee creativity** (Tierney and Farmer, 2011)	EC1	0.812	0.659	0.793	0.866	0.617
	EC2	0.806	0.650			
	EC3	0.783	0.613			
	EC4	0.740	0.548			

*SMC, Square multiple correlation; CR, Composite reliability; AVE, Average variance extracted.*

The results of the discriminant validity test (Fornell and Larcker, 1981) showed that the square roots of average variances extracted (AVE) of model constructs (e.g., CS and EC at the individual level; EB and BMI at the organizational level) were larger than the corresponding cases of interconstruct correlation coefficients (see Table 1), implying that all the measurements presumably possess discriminant validity.

Furthermore, a series of CFAs was employed to assess the distinctiveness of the model constructs. The predicted four-factor model was compared with alternative rival models, including three-factor models and two-factor and one-factor models. Given the correlation among constructs and the data sources of the leader-rated or member-rated models, alternative rival models were constituted by blending the corresponding constructs (see Table 3). The CFA results indicated that the predicted four-factor model showed a much better fit with the data (χ^2^ = 160.66, df = 71; CFI = 0.940; TLI = 0.924; RMSEA = 0.066; SRMR = 0.045) than all possible alternative rival models, demonstrating that all utilized measurements capture adequate discriminant validity and confirm their usefulness for the hypothesis tests.

**TABLE 3:** Results of confirmatory factor analysis.

CFA Model	χ^2^	df	CFI	TLI	RMSEA	SRMR
**One factor model**	897.46	77	0.522	0.436	0.193	0.130
*EB, BMI, CS, and EC were blended*						
**Two factor model**	779.36	76	0.591	0.510	0.180	0.144
*EB, BMI, and CS were blended*						
**Two factor model**	849.52	76	0.550	0.461	0.188	0.127
*EB, BMI, and EC were blended*						
**Three factor model**	492.76	74	0.722	0.658	0.140	0.095
*EB and BMI were blended*						
**Three factor model**	402.87	74	0.782	0.731	0.124	0.126
*EB and CS were blended*						
**Three factor model**	541.80	74	0.728	0.665	0.148	0.126
*EB and EC were blended*						
**Three factor model**	184.87	74	0.935	0.921	0.072	0.047
*CS and EC were blended*						
**Four factor model**	160.66	71	0.940	0.924	0.066	0.045

*χ^2^, chi-square value; df, degree of freedom; CFI, confirmatory fit indices; TLI, Tucker-Lewis index; RMSEA, root mean square error of approximation; SRMR, standardized root mean square residual; EB, entrepreneurial bricolage; BMI, business model innovation; CS, creative self-efficacy; EC, employee creativity.*

### Hypothesis Testing

Multilevel structural equation modeling was applied to assess our proposed multilevel mediation model and simultaneously test both direct and indirect effects. Our proposed model showed an adequate overall fit (χ^2^ = 98.71, df = 52, CFI = 0.941, TLI = 0.914, RMSEA = 0.046, SRMR_with_ = 0.038, SRMR_between_ = 0.045).

As shown in Table 4, all the proposed direct and indirect effects were checked. For direct effects, path modeling showed a significant positive relationship between EB and BMI (β = 0.443, *p* < 0.001), thus supporting Hypothesis 1; a significant positive relationship between BMI and CS (β = 0.194, *p* < 0.05), thus supporting Hypothesis 2; and a significant positive relationship between CS and EC (β = 0.787, *p* < 0.001), thus supporting Hypothesis 3. Interestingly, the results indicated that EB (β = 0.102, *p* > 0.05; β = 0.021, *p* > 0.05) was not significantly related to either CS or EC. Furthermore, BMI (β = 0.208, *p* > 0.05) was not significantly positively related to EC.

**TABLE 4:** Tests of direct and indirect relationships (Hypotheses 1–4).

Path	Estimates	S.E.	Lower and upper 95% CI limits
***Test of direct relationships***			
Top-down direct path (2-1)			
Entrepreneurial bricolage → creative self-efficacy	0.102	0.086	[−0.067, 0.270]
Entrepreneurial bricolage → employee creativity	0.021	0.077	[−0.130, 0.171]
Business model innovation → creative self-efficacy	0.194*	0.084	[0.029, 0.359]
Business model innovation → employee creativity	0.208	0.173	[−0.547, 0.131]
Direct path (1-1)			
Creative self-efficacy → employee creativity	0.787***	0.134	[0.525, 1.050]
Direct path (2-2)			
Entrepreneurial bricolage → business model innovation	0.443***	0.089	[0.268, 0.618]
***Test of indirect relationships***			
Indirect paths model (2-2-1)			
Entrepreneurial bricolage → business model innovation→ employee creativity	0.092	0.073	[−0.236, 0.052]
Indirect paths model (2-1-1)			
Entrepreneurial bricolage → creative self-efficacy→ employee creativity	0.080	0.066	[−0.049, 0.209]
Complete indirect paths model (2-2-1-1)			
Entrepreneurial team knowledge diversity → knowledge sharing → team member creativity → team creativity	0.067*	0.035	[0.010, 0.147]

*For direct relationships (upper panel) and indirect relationships (lower panel), unstandardized estimates are reported. 1, level-1 variable; 2, level-2 variable; CI, confidence interval. Significant direct and indirect effects using Monte Carlo confidence intervals.*

*p < 0.05.

***p < 0.001.

Next, regarding the indirect effects, the multilevel mediating effect of BMI at the organizational level and CS at the individual level on the relationship between organizational-level EB and individual-level EC (i.e., 2-2-1-1 model) was tested. In the proposed multilevel mediation model, all the paths were simultaneously evaluated by the MSEM approach. In particular, the cross-level indirect effects were assessed at the between-organizational level when the proposed model involved the downward effect (Preacher et al., 2010).

Following the recommendation of MacKinnon et al. (2002), the product of coefficients of the independent variable and mediator was calculated to test the multilevel indirect effect. If the product of coefficients was statistically significant, the multilevel indirect effect could be confirmed. In addition, according to the recommendation of Preacher et al. (2010), based on bias-corrected Monte Carlo parametric bootstrapping with 20,000 resamples, a 95% confidence interval (CI) was created to recheck the multilevel indirect effect. The results showed that the cross-level indirect effect of EB on EC via BMI was not significant (unstandardized estimate of the product of coefficients = 0.092, *p* > 0.05), and the 95% bias-corrected Monte Carlo parametric bootstrap confidence interval (CI) around the indirect effect included zero (CI = [−0.236, 0.052]); thus, Hypothesis 4 was not supported.

The cross-level indirect effect of EB on EC via CS was not significant (unstandardized estimate of the product of coefficients = 0.080, *p* > 0.05), and the 95% bias-corrected Monte Carlo parametric bootstrap confidence interval (CI) included zero (CI = [−0.049, 0.209]); thus, Hypothesis 5 was not supported. Furthermore, the cross-level indirect effect of EB on EC via the chain of BMI and CS was significant (unstandardized estimate of the product of coefficients = 0.067, *p* < 0.05), and the 95% bias-corrected Monte Carlo parametric bootstrap confidence interval (CI) excluded zero (CI = [0.010, 0.147]), thereby supporting Hypothesis 6.

**FIGURE 2:**
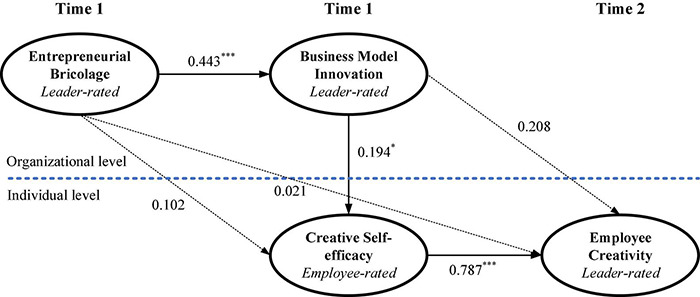
Results of multilevel sequential mediation analysis. *p < 0.05, ^***^p < 0.01; Unstandardized coefficients are presented. Solid arrows represent statistically significant paths, whereas dotted arrows represent statistically nonsignificant paths.

Moreover, according to the recommendation of De Wulf et al. (2001), a comparison between the proposed multilevel model and the alternative rival model (without a direct path, here EB → EC) was carried out on the basis of model fit indices and the proportion of statistically significant paths to examine the full versus partial mediation prediction in this study. The results indicated that the alternative rival model (χ^2^ = 99.29, df = 53, CFI = 0.942, TLI = 0.916, RMSEA = 0.045, SRMR_with_ = 0.038, SRMR_between_ = 0.045) failed to possess an improved model fit, which was verified by a scaled chi-square difference test [Δχ^2^_scaled_ (1) = 0.01, *p* = n.s.]. Hence, it offered evidence for the full mediation hypothesis. The results of the multilevel sequential mediation analysis are shown in Figure 2.

## Discussion

This study examined whether organizational-level EB and BMI have cross-level influences on individual creative behaviors by adopting a 2-wave longitudinal design and collecting data from a sample of 336 employees nested within 49 entrepreneurial internet firms in China. Based on a trickle-down model, this study specifically addressed and analyzed how the cross-level effect of EB flows through organizational-level BMI and individual-level CS to subsequently stimulate employee creativity. In line with our research hypotheses, which drew from social learning theory and social cognitive theory, our findings showed that EB has a cross-level effect on employee creativity, an effect sequentially transmitted through organizational-level BMI and individual-level CS. Our results provide empirical evidence to support the idea that organizational-level EB and BMI are critical for individual creative behaviors. Interestingly, the cross-level influences of these contextual factors (e.g., EB and BMI) on EC mostly rely on the within-level mediating effect of CS.

### Research Implications

This study makes several theoretical contributions to the literature. First, it contributes to the EB and creativity literature by directly and rigorously exploring the EB-creativity link. Given that EB could produce new knowledge of resources at hand (Andersen, 2008; Boxenbaum and Rouleau, 2011), this study concludes that the new knowledge generated in the bricolage process is likely beneficial to individual creative behaviors; a conclusion that complements the current EB literature. Our findings revealed that organizational variables (e.g., EB) are also key predictors of individual creativity; however, prior research has primarily focused on leadership styles (e.g., transformational, authentic, or ethical leadership) within EC (Hennessey and Amabile, 2010). To the best of our knowledge, this study provides the first empirical evidence to verify the effect of EB on individual creative behaviors. In doing so, the present study, regarding the potential influence of EB on individual creative behaviors, contributes to the theoretical understanding and growing recognition of EB as a specific contextual factor that could nurture workforce creativity (Mainemelis et al., 2015). Additionally, prior research, concerned with creative outcomes, has considered creative thinking an outcome variable (Palanski and Vogelgesang, 2011). However, because creative thinking, akin to a trait, is not similar to actual creativity, which is more like a state, it is inappropriate use creative thinking to explore the relationship between EB and creativity. Hence, it is necessary to examine the EB-creativity link by directly measuring actual creativity. To fill this empirical research gap, this study explored the relationship between EB and EC by adopting a validated leader-rated creativity measure.

Second, this study may contribute to the literature on the trickle-down effects of EB and BMI in that our findings indicated that the effects of EB and BMI flow from the organizational level down to the individual level and subsequently manifest in EC. Because prior studies have examined the single-level effects of organizational variables (e.g., team composition variables) on organizational outcomes by predominantly relying on a single-level approach (e.g., Halfhill et al., 2005; Bell, 2007; LePine et al., 2011), few works have explored the cross-level influences of organizational variables on distant followers’ behaviors to fully test the trickle-down model (Kozlowski and Klein, 2000; Simons et al., 2007; LePine et al., 2011). Indeed, Mayer et al. (2009) contended that there is empirical evidence to support the cascading effect of role modeling from high-level leaders on their followers’ responses. Similarly, given that EB and BMI could be regarded as contextual factors at the organizational level, the current research did not directly and rigorously explore and test the relationship between organizational-level EB/BMI and individual-level creativity. As such, as EB and BMI signal that new ideas and new ways of doing things are sponsored in an organizational context, they might also facilitate individual creative behaviors. By extending previous research on how organizational or team-level properties lead to individual-level creativity (e.g., Hirst et al., 2009; Liu et al., 2011; Chen et al., 2013), our findings supported this reasoning, i.e., EB and BMI exerted a top-down effect on individual creative behaviors. Furthermore, prior studies have failed to clearly describe the underlying mechanism in the relationship between EB and EC. According to Guo et al. (2016), a positive relationship between EB and BMI has been identified, but few studies have explored whether EB affects EC through its influence on BMI. As such, the present study is the first empirical research to illuminate the underlying mechanism through which EB can affect EC, thereby contributing to the creativity literature by revealing a clearer picture of the cross-level mediating role of BMI in the EB-EC link.

Third, this study explored the cross-level mediating role of CS in the EB-creativity link. By applying SCT in research on the EB-creativity link, our results, regarding the benefits of EB for CS, test the effect of EB on motivating employees to enhance their beliefs in creative efficacy. According to our results (see Table 4), the cross-level indirect effects of EB on EC via BMI and EB on EC via CS were not significant; however, the cross-level indirect effect of EB on EC, via the chain of BMI and CS, was significant, demonstrating that EB exerted a cross-level influence on EC that is sequentially transmitted through between-level BMI and within-level CS. By highlighting how CS mediates the EB-creativity link, our findings suggest that the cross-level influence of EB, by enhancing BMI, could generate effective motivation for employees and then drive creative performance by building their confidence (Liu et al., 2016). As such, our results stress the significant role of EB in building employees’ beliefs in their own skills and capabilities, in terms of creativity, and subsequently, nurturing EC. Unfortunately, empirical research on this topic is still scarce. By establishing the contribution of EB and BMI to individual creativity-specific efficacy, our findings reveal the underlying motivational mechanism in EB-creativity link research. Hence, our findings provide evidence for whether EB and BMI influence CS through organizational dynamics, and subsequently, promote individual creative behaviors. Furthermore, these findings also indicate that the relationship between EB and EC is complex and cannot be fully disclosed without considering the within-level mediating role of CS. As such, a single-level approach might limit an effective understanding of the trickle-down effects of EB and BMI. By adopting a cross-level approach, the complexity and richness of the implications of EB and BMI across levels of analyses in an organization could be fully captured.

Finally, due to our survey mainly involving entrepreneurial internet firms, this study contributes to EB research by indicating that EB is beneficial to both the organizational performance (i.e., BMI) of and the individual performance (i.e., EC) within internet ventures. Traditionally, EB has been explored in the contexts of ventures in all industries (Baker and Nelson, 2005; Senyard et al., 2014). Among new ventures, in particular, internet ventures are more likely to engage in EB due to the struggle to overcome the constraints of scant resources. However, existing research on the impacts of EB on entrepreneurial internet firms is still limited. Our findings show that EB plays a substantial role in the context of internet ventures. EB, which could be the source of a firm’s accumulated knowledge base (Duymedjian and Rüling, 2010), enables internet ventures to learn from resource recombination actions and enhance creative performance. In line with this reasoning, rather than being regarded as a one-time coping mechanism, EB might actually have a long-term effect on organizations (Ferneley and Bell, 2006).

### Practical Implications

This study provides several potential practical implications. First, given the trickle-down effects of EB on fostering EC, venture firms should attempt to expand EB throughout their organizations. More specifically, our findings showed that EB could not only overcome resource constraints but also proactively facilitate innovation and creativity in entrepreneurial firms. Hence, it is especially advisable for CEOs of venture firms to leverage EB activities in their organizations to motivate employees to generate creative initiatives. Moreover, to better understand the potential uses of resources, it is recommended for leaders of venture firms to host brainstorming conferences to motivate employees to discover the potential opportunities that can emerge in the process of bricolage, which they might then exploit in the near future. In addition, a formal award system is recommended to encourage employees to identify potential opportunities in applying organizational resources, such as how to modify the current use of venture resources to upgrade existing and create more valuable products or services, how to recombine venture resources to capture emerging markets, and how to discover complementary resources to develop new products or services.

Second, our findings regarding the trickle-down effects of BMI showed that BMI plays a mediating role in the link between EB and EC. Therefore, a venture firm should be concerned with the effect of BMI when translating EB activities into employees’ creative behaviors. Thus, with the aim of promoting BMI, a venture firm needs to align incentives with explorations, encourage experimentations with more alternatives, adopt new ways to do business, motivate employees to take risks, and actively commit to applying the combinations of resources at hand to new problems and opportunities (Baker and Nelson, 2005).

Third, that employees’ CS is the key underlying mechanism within the cross-level relationship between EB and EC implies that CEOs of venture firms are key to fostering and enhancing efficacy beliefs about creative capability among their employees. As such, leaders of venture firms should drive expectations and share visions for creative performance to help their employees build strong beliefs about their creative capabilities and full confidence in realizing their creative goals. Leaders of venture firms can also be instrumental by openly expressing confidence in their employees and mobilizing appropriate organizational resources to support them in engaging in creative activities. Furthermore, customized training and individualized coaching on a regular basis should be provided to employees, who will then act as catalysts to improve their skills, upgrade their working approaches, and adapt their creative potentials and beliefs to solve routine problems in creative ways.

### Limitations and Future Directions

Despite possessing several strengths, this study still has some limitations that provide clues for future research. First, although our findings support our prediction that EB influences BMI and then manifests in EC via CS, to draw consistent conclusions about the cause–effect relationship between the relevant predictors and outcomes, it is recommended that future research replicates our study by using quasi-experimental or experimental designs.

Second, given that SLT was applied to interpret the trickle-down effects of EB, the modeling variables could not be measured directly. Future research could expand our study by directly measuring the mechanism variables to evaluate the explanatory power of SLT.

Third, although there are two patterns of bricolage, namely, parallel and selective bricolage (Baker and Nelson, 2005; Senyard et al., 2014), this study examined only the general effect of bricolage without considering the different patterns. Parallel bricolage refers to multiple bricolage projects that can lead to self-reinforcing cycles by collecting and storing diverse materials. In contrast, selective bricolage indicates that bricolage activities are limited to a few domains. According to Rönkkö et al. (2013), the two patterns of bricolage might exert different impacts on organizational outcomes. Future research could extend this inference by examining the different effects of the two patterns on EC.

Fourth, to better understand the cross-level relationship between EB and EC, this study was limited to one organizational-level mediating mechanism (i.e., BMI) and one individual-level underlying mechanism (i.e., CS). Future studies may examine other possible mediating mechanisms informing this link; for instance, the mediating effects of psychological safety (Palanski and Vogelgesang, 2011) and leader trust (Kannan-Narasimhan and Lawrence, 2012).

Finally, our results are based on survey dataset from entrepreneurial internet firms in China, which might not be easily generalized to other economies (Ahlstrom and Ding, 2014). Hence, future researchers could further warrant whether the findings can be transferable to other emerging economies and developed economies in the contexts of various institutional and governance regimes (Yu et al., 2020).

## Conclusion

In today’s knowledge economy era, venture firms have begun to pay more attention to innovation and creativity, especially entrepreneurial internet firms. By applying a 2-wave longitudinal research design and collecting data from multiple sources (i.e., leaders and employees), our findings, based on the trickle-down model, revealed that organizational-level EB activities could foster individual-level employees’ creative performance. More specifically, our results showed that the trickle-down effect of EB may flow, from top to bottom, across organization levels by mediating the effects of BMI at the organizational level and CS at the individual level. Thus, this study enriches the creativity literature and improves the understanding of the value of EB and BMI by extending their implications to EC. Accordingly, this study has valuable implications for both theory and practice.

## Data Availability Statement

The raw data supporting the conclusions of this article will be made available by the authors, without undue reservation.

## Ethics Statement

An ethics approval was not required as per applicable institutional and national guidelines and regulations. The informed consent of the participants was implied through survey completion.

## Author Contributions

FH took charge of the research design, methodology, literature review, analysis and interpretation of data, as well as drafting and revising the manuscript. M-DQ conceived the literature review, research design, and data collection. YS contributed to the literature review, research design, and practice implication. X-XT and B-XY conceived the literature review, practice implication, and data collection. All authors contributed to the article and approved the submitted version.

## Conflict of Interest

The authors declare that the research was conducted in the absence of any commercial or financial relationships that could be construed as a potential conflict of interest.

## Publisher’s Note

All claims expressed in this article are solely those of the authors and do not necessarily represent those of their affiliated organizations, or those of the publisher, the editors and the reviewers. Any product that may be evaluated in this article, or claim that may be made by its manufacturer, is not guaranteed or endorsed by the publisher.
